# Modification of Synaptic-Input Clustering by Intrinsic Excitability Plasticity on Cerebellar Purkinje Cell Dendrites

**DOI:** 10.1523/JNEUROSCI.3211-18.2019

**Published:** 2020-01-08

**Authors:** Gen Ohtsuki

**Affiliations:** ^1^The Hakubi Center for Advanced Research, Kyoto University, Yoshida, Sakyo-ward, Kyoto 606-8501, Japan, and; ^2^Department of Biophysics, Kyoto University Graduate School of Science, Kitashirakawa-Oiwake-cho, Sakyo-ward, Kyoto 606-8224, Japan

**Keywords:** branch-specific clustering, cerebellar Purkinje cells, dendrites, intrinsic plasticity, SK channels, synaptic currents

## Abstract

The role of dendrites in the integration of widespread synaptic activity has been studied in experiments and theories (Johnston et al., 1996; Magee, 2007). However, whether the conduction of synaptic currents from dendrites to the soma depends on excitability of those dendritic branches is unclear. How modulation of the branch excitability affects the conduction of synaptic inputs and their selection on dendrites is also elusive. Here, I performed simultaneous voltage-clamp recordings from the soma and dendrites of single cerebellar Purkinje neurons in male Sprague-Dawley rats and analyzed the relationship between spontaneous EPSCs on both sides. I found that EPSCs on distal dendrites have a salient discordance in amplitude compared with those on the soma. Furthermore, individual ratios of the EPSC concurrently recorded on the soma and dendrites were not unique, but discrete, suggesting the occurrence of various attenuations in different paths of dendritic branches to the soma. The obtained data and simulations indicate several distinct groups (4.5 ± 0.3, *n* = 22 somatodendritic recordings) of co-occurred synaptic inputs in Purkinje cell dendrites. This clustering of synaptic currents was suggested to emerge at farther distances than the secondary bifurcations. Finally, ratios of the co-EPSCs were uniformly distributed after either intrinsic plasticity induction or SK-channel blockade. Overall, results suggest that in Purkinje cells the excitability along the dendrite processes modulates the conduction of EPSCs and makes active inputs heterogeneous through SK channel activity, intrinsic plasticity, and dendritic branching. These properties of dendrites may confer branch-specific computational power to neurons.

**SIGNIFICANCE STATEMENT** I have previously studied the “non-synaptic” plasticity of the intrinsic excitability in the cerebellar Purkinje cells (Belmeguenai et al., 2010), and branch-specific increase of intrinsic excitability of the dendrites (Ohtsuki et al., 2012b; Ohtsuki and Hansel, 2018) through the downregulation of SK (small conductance Ca^2+^-activated K^+^) channels. In this study, I show that a dendritic filtering of synaptic electroconductivity is heterogeneous among the branches on distal dendrites and that the increase in the dendritic excitability accompanied with the intrinsic plasticity alters a state with the heterogeneity to a globally excitable state in Purkinje neurons. My findings propose a new learning model relying on the intrinsic excitability plasticity of the dendritic branch fields.

## Introduction

Depolarization of postsynaptic membrane is primarily integrated at the dendrites, and the voltage changes transmit to the soma as a determinant of spike generation. In the cerebellum, Purkinje cells (PCs) are the principal output neurons influencing many aspects of the activity-dependent modification of the intrinsic membrane properties on the spike patterns (Belmeguenai et al., 2010; Ohtsuki et al., 2012b; Grasselli et al., 2016; Ohtsuki and Hansel, 2018). Intrinsic plasticity (IP), as an increase in the intrinsic membrane excitability, is induced by either transient depolarization on soma or parallel fiber (PF) stimulation via both the phosphatase-dependent signaling and the downregulation of small conductance Ca^2+^-activated K^+^ channels (SK channels). Originally, Schreurs et al. (1998) reported the enhancement of excitability of PC dendrites following the classical conditioning of rabbit eyelid responses. The threshold of the dendritic action potential *ex vivo* was reduced after 3 d of classical conditioning, which suggests that the excitability of neuronal membrane, including dendrites, was modulated in association with learning. Based on the observations in conditional protein phosphatase 2B (PP2B) knock-out mice which are deficient in the molecule specifically in the PCs, this IP was associated with the cerebellar motor coordination learning of eye movements and the delay eye-blink conditioning (Schonewille et al., 2010). Recent finding suggests that activated microglia induce the IP of the PCs. The microglia-triggered hyperexcitability in the cerebellum modulates rodents' psychomotor behaviors, which expressed depression- or autistic-like phenotypes (Yamamoto et al., 2019). Moreover, IP also reduces spike-pause periods through a reduction of the afterhyperpolarization (Grasselli et al., 2016). Such decrease in the spike-pause periods of PCs may affect the time-rocked spike firings in both the cortical PCs and nuclei neurons (Person and Raman, 2011; Brown and Raman, 2018).

IP has been found in many types of neurons (Hansel et al., 2001; Daoudal and Debanne, 2003; Zhang and Linden, 2003; Magee and Johnston, 2005). In other regions of brain, blockade of SK channels potentially unmasks the effect of postsynaptic shunt, following depolarization and elevation of internal Ca^2+^ concentration (Seutin et al., 1993; Faber et al., 2005; Ngo-Anh et al., 2005; Faber, 2010). Considering that a single synaptic transmission has a minor impact on voltage changes of the membrane, the efficacy of the long-term potentiation (LTP) and depression on the initiation of an action potential may be lower than IP, which directly modulates the firing pattern (Ohtsuki and Hansel, 2018). Rather, this type of “non-synaptic” plasticity has a considerable potential for regulating the conduction of postsynaptic currents on dendrites. However, at a cellular level, it is yet to be understood whether IP contributes to the electroconduction of synaptic activities.

It is known that all voltage changes on the distal dendrites may not transmit to the soma (Isope and Barbour, 2002). In PCs, ∼85% of PF synapses do not generate detectable electrical responses at soma, indicating that there is a certain mechanism that enables the passage of active local-voltage changes through PC dendrites. Recently, we showed that the IP of cerebellar PCs (i.e., the modulation of the intrinsic membrane property inherited by SK channels and the counterpart of SK channel downregulation) is a crucial factor for spike generation (Ohtsuki and Hansel, 2018). Importantly, it was possible to induce a branch-specific modulation of the dendritic excitability and complex spikes by simultaneous multiple recordings (Ohtsuki et al., 2012b). Additionally, non-uniform expression pattern of the SK2 channel molecules on dendrites, which is dominantly expressed among the SK channel superfamily in PCs, appears biased among the branches (Belmeguenai et al., 2010, their Fig. 4); therefore, a distinct physiological function was speculated. In the present study, I examined whether PC dendrites can drive the afferent inputs via branch-specific modulation of passive conduction. Further, if synaptic activity itself suppresses the conduction of synaptically-evoked currents on the dendritic processes, the branch field should make active and inactive forms of the functional compartment on neurons. Therefore, IP induction on the global dendritic-field may unleash the filtering effect.

## Materials and Methods

All procedures were performed in accordance with the guidelines of the Animal Care and Use Committees and approved by the Ethical Committee of the local institutions (the University of Chicago and Kyoto University). All animal handling and reporting comply with the ARRIVE guidelines.

### 

#### 

##### Patch-clamp recordings.

*In vitro* patch-clamp recordings were obtained as described previously (Belmeguenai et al., 2010; Ohtsuki et al., 2012b). Sagittal slices of the cerebellar vermis (250 μm) were prepared from male Sprague-Dawley rats [postnatal day (P)24–P33] after administration of isoflurane anesthesia and decapitation. Subsequently, the slices were cut on a vibratome (Dosaka EM) using ceramic blades and kept in artificial CSF (ACSF) containing the following (in mm): 124 NaCl, 5 KCl, 1.25 Na_2_HPO_4_, 2 MgSO_4_, 2 CaCl_2_, 26 NaHCO_3_, and 10 _D_-glucose, bubbled with 95% O_2_ and 5% CO_2_. During cutting, supplemental ingredients (5 mm Na-ascorbate, 2 mm thiourea, and 3 mm Na-pyruvate) were added to the ACSF. After at least 1 h, slices were transferred to a recording chamber which was superfused with ACSF and at a near-physiological temperature (31–34°C), which was supplemented with 100 μm picrotoxin to block GABA_A_ receptors. The patch-clamp recordings were performed under a 40× water-immersion objective lens equipped with a DIC system (DS-Qi2; Nikon) mounted on a microscope (ECLIPSE FN1, Nikon), whereas recordings were performed in the voltage-clamp or current-clamp mode using an EPC-10 amplifier (HEKA Elektronik). Both membrane voltage and current were filtered at 2.9 kHz, digitized at 10 kHz, and acquired using Patchmaster software (HEKA Elektronik). Patch pipettes (borosilicate glass) were filled with either K-gluconate or Cs-gluconate solution. K-gluconate solution contained the following (in mm): 9 KCl, 10 KOH, 120 K-gluconate, 3.48 MgCl_2_, 4 NaCl, 10 HEPES, 4 Na_2_ATP, 0.4 Na_3_GTP, and 17.5 sucrose, pH 7.25 titrated with 1 m KOH. Cs-gluconate solution contained the following (in mm): 9 CsCl, 130 CsOH, 100 d-gluconic acid, 3.48 MgCl_2_, 4 NaCl, 10 HEPES, 4 Na_2_ATP, 0.4 Na_3_GTP, and 17.5 sucrose, pH 7.25 titrated with 2.6 m CsOH. The membrane voltage was offset for liquid junction potentials (11.7 mV for K-gluconate solution; 12.5 mV for Cs-gluconate solution). The somatic patch electrodes had electrode resistances of 2–4 MΩ, whereas the dendritic ones had electrode resistances of 5–8 MΩ (Ohtsuki et al., 2012b). Hyperpolarizing bias currents (100–400 pA) were injected to both stabilize the somatic membrane potential at ∼−71.7 mV and to prevent spontaneous spike activity.

Series resistance was monitored by injecting 50 pA hyperpolarization pulses on soma and dendrite, respectively. In each trial of hyperpolarization, the measurement of passive membrane current in dendrite and soma proved both recordings to be from identical neurons. Data were collected both at the soma and at primary to tertiary branches or more at a distance of 39–155 μm apart from the soma (*n* = 53 recordings from 47 cells). Furthermore, different-cell pairs at a distance of 41–205 μm were recorded in some experiments (*n* = 15 pairs). During the recording of spontaneous EPSC (sEPSC) events, the membrane current was maintained at either −71.7 or −81.7 mV (only when the membrane current was jittered). Periods of fluctuation were omitted and supplemented by other trials. Total durations of the paired-recordings were variable, due to the broad ranged frequency of sEPSC (6.2 ± 5.2 Hz, mean ± SD; 0.5–21.6 Hz, *n* = 35). I analyzed data from 1 to 15 min periods, depending on the sEPSC frequency. The data were arbitrarily separated to two groups: the proximal and distal dendrites at farther than 75 μm, whose border was determined from following reasons; (1) one-third of the whole length of PC dendrite, corresponding to the border between CF and PF projections; (2) the location which was typically a secondary or tertiary bifurcation according to the morphological data (see Fig. 2); (3) the distinct differences in the level of sEPSC discordance (see Fig. 4*I*). In several somatodendritic recordings, *post hoc* histology of the recorded neurons was performed (*n* = 8 cells). All stained cells were revealed to locate at the straight layer of the lobule (Fig. 2). Recordings were done from randomly selected lobules (lobules II–VIII). In several experiments, I recorded sEPSCs for at least 20 min after the applying either the induction protocol or the drug administration in experiments involving IP induction and SK-channel blockade. Depolarizing pulses (300–480 pA/100 ms) were applied on the soma at 5 Hz for 3–4 s for IP induction, as the conditioning protocol. Apamin (10 nm; Tocris Bioscience, R&D Systems, catalog #1652) was applied to the bath chamber through a circulation system. When recording with the cesium-containing internal solution, the invasion of solution to the dendritic process was waited for >5 min after membrane break.

In cerebellar Purkinje-cell dendrites, while climbing fiber terminals form synapses on the proximal dendrite (one-third of whole length of ∼240 μm), PF terminals form synapses on the distal dendrite (Ichikawa et al., 2016). In addition, the ascending axons of granule cells were also known as another group projecting to the PCs as a source of sEPSCs (Llinas, 1982), implying a distinct function. Gundappa-Sulur et al. (1999) gave a percentage of ascending segment synapses ranging between 7 and 24% of the total in mature female rats. The presynaptic volume of varicosities is 35% larger in ascending segment than in parallel fibers. Although the difference in EPSC amplitude was not discernible between proximal ascending axon and parallel fiber, proximal granule cells had much higher release probability (Isope and Barbour, 2002). Here, the percentage of ascending connections included in the spontaneous EPSCs could not be determined. Regarding postsynaptic expression of the AMPA receptors, it is yet unclear whether postsynaptic conductance of glutamatergic receptors has a property of progressively increasing their expression at the relevant dendrites.

##### *Post hoc* morphology and imaging.

To identify the morphological relevance to the functional clustering on the PC dendrites and regional correlation among lobules in the cerebellar cortex, I performed the reconstruction of the cellular morphology in several recordings. Cells were infused with 0.5% Neurobiotin (SP-1120, Vector Laboratories) or biocytin (Sigma-Aldrich) from both electrodes. Sucrose of the internal saline was reduced and substituted to the Neurobiotin or biocytin. The osmolarity of the final internal saline was carefully adjusted to 300–305 mOsm/kg for soma and 290–295 mOsm/kg for dendrite with additional sucrose. When using Neurobiotin, which is negatively charged, sustained depolarization to 0 mV were applied at least for 10 min after whole-cell recordings to increase permeability of the substance. After experiments, the slices were fixed in 4% paraformaldehyde for 48–72 h. Biotin-filled cells were visualized using standard procedures of *post hoc* staining. Fixed slices were rinsed with PBS three times. Cells were permeabilized with 1% Triton X-100/PBS for 60 min at room temperature and rinsed with PBS. The slices were then treated with ABC Vectastain Kit (Elite Pk-6100 Standard, Vector Laboratories) for 2 h and rinsed. Sigma-Fast DAB (Sigma-Aldrich) with 0.3% CoCl_2_ (w/v) was dropped to a slice to visualize the cell morphology. I used RapiClear (1.47 or 1.49; SunJin Lab) to mount the slices on the glass slides for tissue clearing. PCs were monitored on a FV1000 BX61 confocal microscope with FluoView software (Olympus). Images were acquired at a resolution of 512 × 512 FOV with a *z*-step size of 0.2–0.3 μm. Branch length of dendrites was measured with ImageJ and Fiji software (NIH).

##### Data analysis.

Data were analyzed using a custom program written in MATLAB (MathWorks; RRID:SCR_001622). For the analysis of sEPSC events, an FFT bandpass (1–90 Hz) and a Savitzky–Golay filter (*smooth* function with Savitzky–Golay filter with 8° of a polynomial model) were applied to the recorded currents. In the event detection, threshold for sEPSCs applied to the filtered traces was set at 2 pA. Events were defined as those exceeding 3.5 times the SD during the 4 ms pre-period, whereas the peak was detected within 10 ms after the initiation of an EPSC. Subsequently, I applied the *fminsearch* function to obtain the decay time of a single exponential. When the current trace at the decay period (limited to 19 ms) fit poorly, the data were excluded. The rise time was regarded as the period spanning between the 10–90% of the change from the peak to the basement values. Rise time and half-width of sEPSCs were not significantly different between soma and dendrite except for apamin-administration (Table 1). Figures 3, *A* and *B*, and 4*A–C* report the sample traces to which a combination of FFT bandpass and Savitzky–Golay filters was applied. In the representative waveform traces of sEPSC in Figure 3*C*, 221 events from the soma and 171 events from distal dendrite were averaged, whereas in 3*D*, 218 events from the soma and 153 events from the proximal dendrite were averaged. Similarly, in Figure 4*D*, 522 events from the soma and 308 events from the distal dendrite after IP-conditioning were averaged, whereas in 4*E*, 123 events from the soma and 89 events from the distal dendrite under SK-channel blockade were averaged. In Figure 4*F*, 436 events from the soma and 496 events from the distal dendrite under broad K-channel blockade by cesium ion were averaged. With regard to the cumulative probability in Figure 3*F*, a maximum of 200 sEPSC amplitudes and frequency events were collected randomly from each cell from each experiment. In this study, the term *discordance* was specifically defined as the absolute difference in EPSC amplitude between the soma and dendrite of the paired recording. Discordance in synaptic current was calculated as the arithmetic mean of the absolute values of every subtraction of each detected sEPSC on the soma and dendrite after normalization (see Figs. 3*G*, 4*H*,*I*), following the equation:

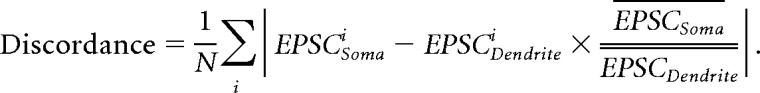
 The extent of the discordance reflects the absolute difference of EPSC amplitude on both sides (see Figs. 3*A*,*B*, 4*A–C*). I did not include one set of data (*X* = 40 μm, Proximal control) because of the background fluctuation of the current only for this analysis. In total, the number of sEPSC events of paired somatodendritic patching is between 313 and 15,533 (*n* = 53). Co-events were determined as the coincident events occurring at two locations within a time window of 6.5 ms. The window for detection was determined from above the value of [(1/2) × half-width of EPSC (full-width at half-maximum [FWHM]); FWHM: 11.4 ± 2.2 ms, mean ± SD, *n* = 26 recordings of control soma and dendrite], which is assumed enough to detect co-EPSC but to fairly exclude duplication. From detected soma- and dendrite-dominant EPSCs, “Soma-only” and “Dendrite-only” events were extracted by the threshold. Remained co-events are “Dendrite and soma-coupled” co-EPSCs. The numbers of detected co-events (Dendrite and soma-coupled) was from 55 to 2337. The proportion of Dendrite and soma-coupled, Dendrite-only, and Soma-only EPSC events are shown in the stacked bar graphs in (see Figs. 6*A–C*, 10*A–C*). The number of the total sEPSC events of different-cell pairs was from 798 to 4270 (*n* = 15), whereas the number of co-events in different-cell pairs was from 26 to 392, which is 5.7 ± 2.4% (*n* = 11 pairs) of the co-EPSCs (Fig. 6*D*,*E*). The coefficient of determination was *R*^2^ 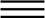 1 − (residual sum of squares/total sum of squares) from the linear regression (see Fig. 6*D*). Regarding two datasets at the borderline between distal and proximal dendrites (*X* = 75 μm), I included the two datasets of distal dendrites because of the low proportion of co-EPSCs (13.5 and 20.2%) compared with other proximal data for the analysis of clustering (see Figs. 6*D*,*E*, 10*G*,*H*).

**Table 1.** Waveform parameters of spontaneous EPSCs.

Control, Distal		Control, Proximal		IP-conditioned		Apamin	
Soma	Dendrite	Soma	Dendrite	Soma	Dendrite	Soma	Dendrite
10–90% rise time							
4.4 ± 0.3 ms	4.5 ± 0.3 ms	4.6 ± 0.2 ms	4.4 ± 0.2 ms	4.4 ± 0.1 ms	4.3 ± 0.3 ms	4.6 ± 0.3 ms	4.8 ± 0.3 ms
*n* = 13 pairs		*n* = 6 pairs		*n* = 8 pairs		*n* = 8 pairs	
*p* = 0.166		*p* = 0.548		*p* = 0.2786		*p* = 0.1304	
FWHM							
12.1 ± 1.8 ms	10.7 ± 2.5 ms	11.4 ± 3.9 ms	9.2 ± 2.9 ms	10.4 ± 3.2 ms	10.1 ± 3.0 ms	13.8 ± 1.1 ms	9.0 ± 3.1 ms
*n* = 13 pairs		*n* = 6 pairs		*n* = 8 pairs		*n* = 8 pairs	
*p* = 0.09		*p* = 0.421		*p* = 0.9591		*p = 0.0019	

The values of 10–90% rise time and FWHM of control, IP-conditioned, and apamin-administrated experiments at soma and dendrite using K-gluconate internal solution are shown as mean ± SD with *p* values by two-tailed Mann–Whitney *U* test.

*Significant difference.

Estimation criteria of the clustering (see Figs. 6*A–C*, 10*A–C*):

1. I set a threshold for the proportion of co-EPSC events at 6%, which indicates that the datasets with <6% were considered coincidental and treated as emerged by chance within the timespan of detection, thus without any clustering. This level is determined from the proportion of co-events in different-cell pairs, 5.7 ± 2.4%. Although the chance level was set at 6% under assumption of non-correlation of sEPSC in different PCs, this assumption is lack in the possibility of correlated PF inputs (i.e., in a case when a PF bundle makes synapses on both different cells which are recorded,) and the estimation neglects the variance of data. As shown in Figure 6*D*, the percentage of co-EPSCs declines through the function of the distance. Therefore, please note that the percentage of co-EPSCs is low >100 μm without IP-conditioning, SK-channel blockade or Cs-internal solution. Namely, in far distal dendrites, it becomes difficult to distinguish the co-EPSCs with different degrees of the D/S ratio from EPSCs, which just occurred by chance. In contrast, IP conditioning, SK-channel blockade, and Cs internal-solution groups show higher co-EPSC percentage at >100 μm, suggesting higher electroconductivity.
2. Next, the angular D/S ratios were obtained by the arctangent of the D/S ratios corrected by the mean EPSC amplitude. I designed a histogram of the angular D/S ratios binned at π/40 radian and identified the primal peak of histogram (see Fig. 7). If any accessory peak is with a gap of >1/3 the height of the main peak within the range of width from π/40 to 3π/20 radian (from 4.5 to 27°), it was identified as the independent peak. The accessory peaks should include >3% of the total number of co-EPSCs. The angular D/S ratios in each peak were considered to correspond to the clustered inputs as proposed in Figure 5*A* (Clustered input). The analysis with the Kernel density estimation also provided similar results (data not shown). I then confirmed that the accessory clusters have a positive-to-negative turn from a monotonous increase/decrease of the number of the angular D/S ratio [i.e., (no. of D/S ratio)*_n_*_+1_ − (no. of D/S ratio)*_n_* as shown in Figs. 7, 10*D–F*, difference sequence of D/S ratio histogram].
3. To avoid the accidental selection of data with several peaks to be chosen by chance, I first requested for the false-negative rate (FNR) of the Δ angular D/S ratio (ΔDSR), which was the absolute difference of all pairs from the angular D/S ratio. Therefore, I prepared the ΔDSR and the shuffled-ΔDSR, which was obtained by shuffling the EPSC_soma_ of co-EPSC events. Subsequently, I repeated 500 trials with the two-sampled Kolmogorov–Smirnov test at the significant level of 0.05. FNR identified the number of trials above the significant level of statics. Last, I took the paired-recording data as those with clustered when FNR < 0.20: i.e., the statistical power (percentage of discarding null hypothesis) was >0.80 (related to Ohtsuki et al. (2012a)). Otherwise, the number of clusters is nulled (*n* = 6 of 68 data).
4. Subsequently, the non-randomness was confirmed via the following criteria: when the data were multimodal until the process (3), it was taken as non-random; when the data presented a single peak, it was estimated by the FNR of the angular DSR by two-sampled Kolmogorov–Smirnov test; and when the FNR of DSR < 0.20 (statistical power > 0.80), the DSR dataset was not obtained by chance. Otherwise, the number of clusters is zeroed (*n* = 5 of 68 data).

##### Experimental design and statistical analysis.

All the data are shown as the mean ± SEM across the group datasets, unless otherwise stated. The complete results of the statistical analyses, including degrees of freedom and estimates of effects size, are reported in the Results. Two-tailed Mann–Whitney *U* test was used to compare the data between the two independent groups in Figures 3, *E* and *G*, and 4*G*. One-way ANOVA with Tukey's honestly significant difference (HSD) procedure for multiple comparison was applied in Figures 4*H*, 6*E*, and 10*H* (at the significant level of *p* < 0.05). Pearson's correlation coefficient and the *p* value in Figure 6*D* were obtained with using a MATLAB function, *corrcoef*. Bar graphs and colored marks represent mean ± SEM. All the statistical analyses were performed using MATLAB.

##### Model.

The concatenated-ball model was considered using MATLAB. To simplify the membrane fields of neuron, I compartmentalized them into multiple-fields, such as S, D1, and D2 in the case of the three-ball model (see Figs. 8, 9). Each field was spherical with a diameter of *l*_S_, *l*_D1_, and *l*_D2_, respectively. It was arranged in tandem with following length constants: λ_S_, λ_D1_, and λ_D2_, respectively. The linear display of the concatenated field with different length constants is shown in Figure 8*B* (0, *D*_max_) (Rall, 1962). Considering 1000 synapses that randomly formed on the entire membrane field, I created random points in 3D coordinates within the spheres of concatenated balls (with the *rand* function using the Mersenne Twister random number generator). EPSC amplitudes on synapses were chosen randomly from the Gaussian distribution within *N*(2σ, σ^2^) (0, EPSC_max_) in each trial. Successively, I requested the EPSC amplitudes on the soma (at 0) and dendrite (at D_max_) via the attenuation through the exponential decay function with the length constant under the assumption of constant voltage (i.e., under the voltage-clamp). While in the one-ball model, I set the following parameters: *D*_max_, EPSC_max_, *l*_S_, λ_S_ = 120 μm, 30 pA, 120 μm, 90 μm, respectively (see Fig. 8*C*); in the two-ball model, *D*_max_, EPSC_max_, *l*_S_, *l*_D_, λ_S_, λ_D_ were 120 μm, 30 pA, 60 μm, 60 μm, 90 μm, 70 μm, respectively; in the three-ball model, *D*_max_, EPSC_max_, *l*_S_, *l*_D1_, *l*_D2_, λ_S_, λ_D1_, λ_D2_ were 120 μm, 30 pA, 40 μm, 40 μm, 40 μm, 90 μm, 70 μm, 60 μm, respectively. To simulate co-EPSCs at adjacent positions (see Fig. 8*D*), the two-ball model with the following parameters was used: *D*_max_, EPSC_max_, *l*_S_, *l*_D_, λ_S_, λ_D_ = 45 μm, 30 pA, 60 μm, 60 μm, 90 μm, 70 μm, respectively. The threshold of simulated EPSC was set at 2 pA. Finally, I repeated the trial for 2000 times, and I obtained both averaged probability of the co-EPSC coordinates and the simulated angular D/S ratio histogram as the summation of repetitions at a bin size of π/40 radian. In Figure 9, various length constant (λ_S_) was applied to the one-ball model. The larger λ_S_ makes the distribution of D/S ratio shaper, in this simple simulation. Please note that the *in silico* simulation does not consider the morphological lean of the PCs against the perpendicular alignment of PF projection, the ablation of PC branches and projection fibers by sectioning. The conditions of the simultaneous patch-clamping were handled equivalently, so that the obtained data showed asymmetric distributions of sEPSCs in Figures 6*A–C* and 10*A* and *B*.

##### Code/software accessibility.

Source data that support the findings of this study and custom MATLAB code for analyses are available from the corresponding author upon individual request.

## Results

### Discordance of synaptic inputs between the soma and dendrites from paired recordings

**Figure 1.**
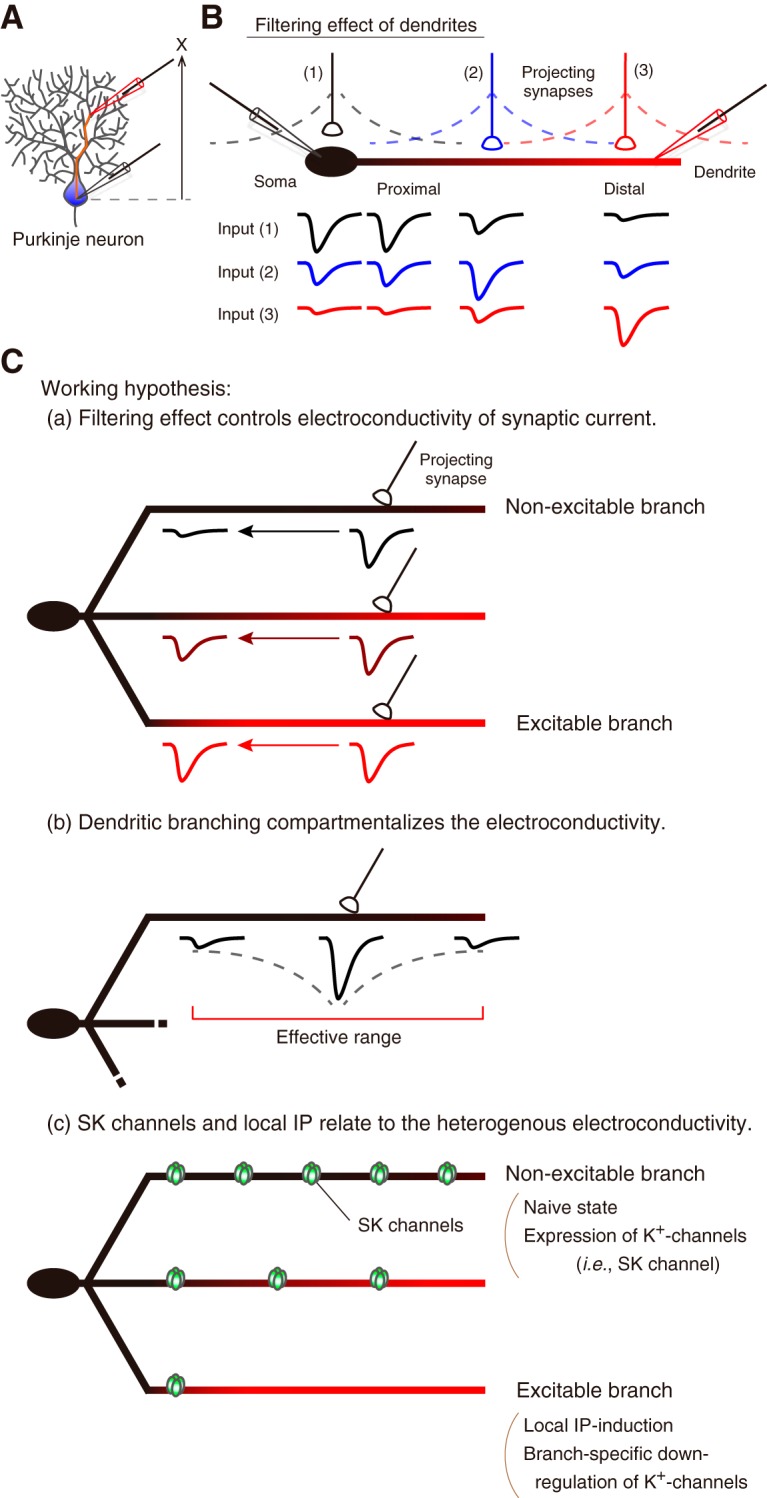
Schema of the synaptic current conduction in the somatodendritic recording. ***A***, A schematic representation of a paired-recording from the soma and dendrite. *X* is the distance of the two recording positions. ***B***, In paired recordings, synaptic currents evoked around the soma (1) are transmitted through the dendrite branch. The EPSC amplitude is attenuated by the membrane capacitance and series resistance. This mechanism is known as the filtering effect of cables. Similarly, synaptic currents evoked around the dendrite (3) also attenuate the amplitude in the opposite direction of the conduction (i.e., dendrite to soma). Synaptic inputs around the proximal dendrite (2) propagate to both the soma and distal dendrite. ***C***, Working hypotheses regarding the conduction of EPSC through PC dendrites: (***Ca***) whether the filtering effect alters the electroconductivity of synaptic current, (***Cb***) whether the branch pattern of PC dendrite affects the conductivity of EPSC, and (***Cc***) whether SK channels and IP make the difference in the conductivity of EPSC. In (***Ca***), EPSC on distal dendrites attenuates through the dendritic process, presumably depending on the excitability of the branch. The EPSC does not conduct farther in the non-excitable dendrite, although the EPSC goes far with less attenuation in the excitable dendrite. Therefore, the electroconductivity of each branch may be heterogeneous. Given that the conduction of EPSC is limited due to the filtering effect and a branch morphology (***Cb***), the EPSC would have an effective range. The dendritic branching may generate substantial compartments of the electroconductivity in PC dendrites. Because the excitability of PC dendrites varies branch-specifically (Ohtsuki et al., 2012b), the heterogeneous conductivity among branches may be produced by downregulation of K^+^ channels (i.e., SK channel) via local IP-induction (***Cc***).

**Figure 2.**
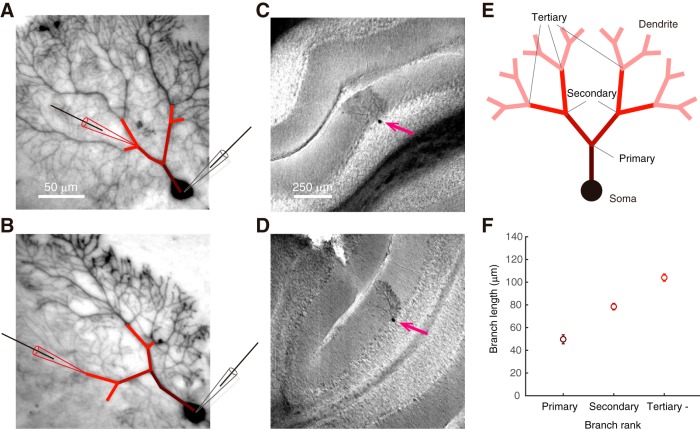
Branch length of the cerebellar PCs in the somatodendritic recording. ***A***, ***B***, Representative histology of the paired recorded PCs. Patch pipettes indicate locations of the patch clamping from soma and dendrite. ***C***, ***D***, Representative images of the stained PCs of (***A***) and (***B***), respectively, at low-magnification. Magenta arrows indicate the location of cell bodies. All stained cells are located at the straight layer of in the lobule. ***E***, A diagram arborization of a PC. Primary, secondary, and tertiary bifurcation points are indicated. Primary branch is between soma and primary bifurcation. Secondary branch is between primary and secondary bifurcations. ***F***, Total branch length to the ranked bifurcations. Through *post hoc* histology images, the length from soma center to bifurcations are collected (*n* = 8 cells). Colored marks with errors represent the mean ± SEM of the ranked branch length.

Paired whole-cell patch-clamp recordings were performed from the soma and dendrites of PCs in cerebellar slices obtained from young adult Sprague-Dawley rats at a near-physiological temperature. To test the scheme of filtering effect (Fig. 1*A*,*B*), I analyzed the EPSC events by paired-recordings. Key questions to reveal here are as follows: (1) whether the filtering effect of PC dendrites alters the conductivity of EPSC; (2) whether dendritic branching and confined electroconduction generates substantial compartments in PC dendrites, and, when existed; (3) whether such heterogeneous electroconductivity among dendrites is regulated by activity-dependent K^+^ channels and plasticity of the membrane excitability; i.e., IP (Fig. 1*C*). Furthermore, in some recordings, I performed *post hoc* histology to investigate the morphological relevance of neurons (Fig. 2). Recorded cells were intact and well arborized from stained Purkinje-cell morphology. Branch length of primary, secondary, and tertiary bifurcations were linearly increased (Fig. 2*F*).

**Figure 3.**
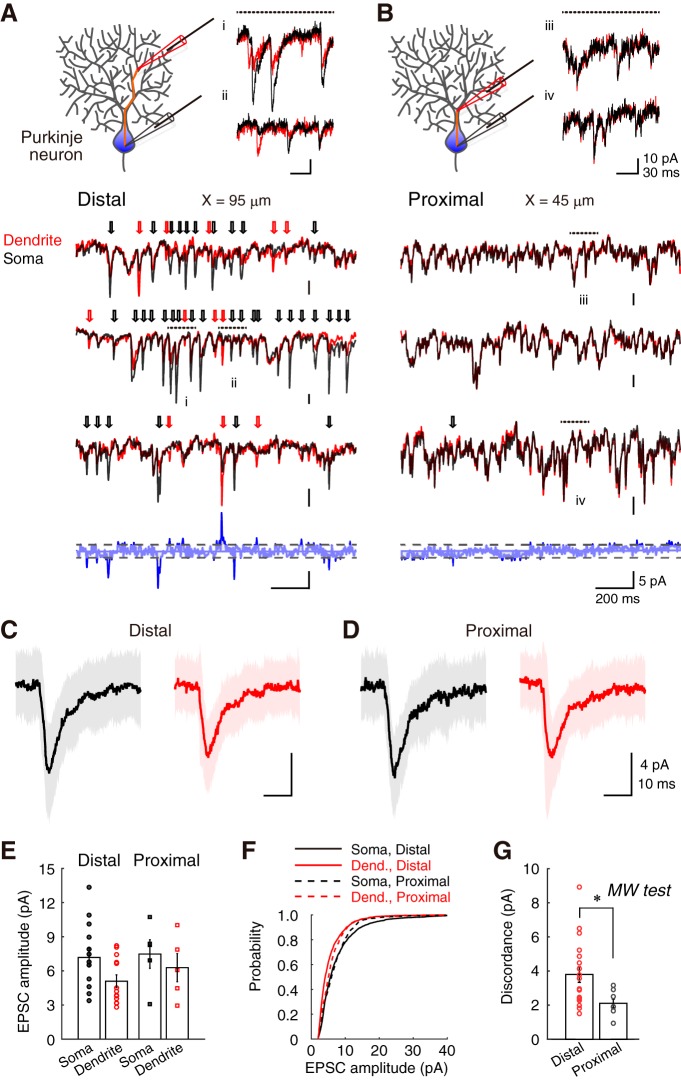
Discordance in the synaptic current of distal dendrites. ***A***, Representative traces of sEPSCs in paired-recordings from soma (black) and distal dendrite (red). Paired patch-clamp configuration is drawn at the top left. Representative raw sEPSC traces are shown at the top right (***Ai***, ***Aii***). Three sample recordings for 1.5 ms are presented in the middle. Black and red arrows indicate soma- and dendrite-dominant events, respectively. Dotted horizontal lines indicate the period of the extracted raw data, associated with ***i*** and ***ii***. Subtraction of the third paired traces is shown at the bottom as blue traces. The detection threshold was drawn as broken gray lines. Please note that there are incongruous co-EPSC events. ***B***, Representative traces of sEPSCs from soma and proximal dendrite. Display style is same as ***A***. ***C***, ***D***, Averaged sEPSCs from soma and either distal- or proximal-dendrite, respectively (black trace, soma; red trace, dendrite). Shading represents the SD (153–221 traces). ***E***, Bar graphs of EPSC amplitudes from individual data (Distal, *n* = 13; Proximal, *n* = 6). ***F***, Cumulative probability of EPSC amplitudes. ***G***, Discordance in sEPSCs compared between distal- and proximal-dendrites. *p < 0.05, two-tailed Mann–Whitney *U* test.

To investigate the activity of functional synapses, I recorded sEPSCs on soma and dendrite simultaneously (Fig. 3*A*,*B*). The distance in patch-clamp location between the dendrites and soma is dependent on the recording (*X* = 39–135 μm). Analysis of sEPSC revealed a discordance in EPSCs between the soma and distal dendrites. In this study, I defined the term *discordance* as the difference between EPSCs on soma and dendrite of the paired recording (see Materials and Methods). Sample recordings are illustrated in Figure 3*A* (*X* = 95 μm; dendrite, red; soma, black), in which EPSC events were either larger on the soma than dendrites (black arrows) or opposite (red arrows). The subtraction of the soma and dendrite sEPSC traces clearly showed the discordance between sEPSC events, implying the existence of soma- and dendrite-dominant events among a series of sEPSCs. Considering that synaptic currents do not substantially attenuate through the short branching paths (Fig. 1*B*), the discordance of sEPSC was expected to be minor in nearby recording positions. As expected, sEPSCs were similar in the proximal dendrites (Fig. 3*B*). The subtraction of sEPSC traces of the soma and proximal dendrites shows smaller differences than those of distal dendrites.

Subsequently, I measured the waveform of each postsynaptic event. The waveforms were not significantly different between paired recordings of distal (*X* ≥ 80 μm) and proximal (*X* ≤ 75 μm) dendrites (Fig. 3*C*,*D*; Table 1). The two regions are separated based on the projection pattern of PF and CF terminals, the secondary branching, and the degree of sEPSC discordance. The EPSC amplitude on dendrite was smaller in the mean amplitude than on soma (Fig. 3*E*,*F*), even though the difference was not of significance (soma vs distal dendrite: rank sum = 214, *n* = 13, *p* = 0.0513; soma vs proximal dendrite: rank sum = 32, *n* = 6, *p* = 0.4206; two-tailed Mann–Whitney *U* test). The difference in EPSC amplitudes should come from the difference in the diameter of patch pipettes and the extent of the membrane break by patch pipettes, reflective of both the thin processes and the difference in series resistance. Because of this reason, EPSC amplitude on dendrites are considered smaller in the entire datasets. However, the discordance of sEPSC (the mean of the absolute difference in sEPSC events between the soma and distal dendrites in paired-recordings; see Materials and Methods) was significantly higher than that from the soma and proximal dendrites (*U* = 10, *n*^Distal^ = 13, *n*^Proximal^ = 6, *p = 0.0092, two-tailed Mann–Whitney *U* test; Fig. 3*G*). Again, although the averaged EPSC amplitude is ∼2 pA larger in soma than in dendrite, the extent of discordance in distal dendrites was ∼4 pA. This suggests that synaptic currents in distal dendrites attenuate through the branch paths in both directions (i.e., from dendrite to soma and from soma to dendrite), and the attenuated EPSCs largely contribute to the increase in the discordance of sEPSC in distal dendrites (Fig. 1*B*).

### Intrinsic plasticity and SK-channel blockade diminished the EPSCs discordance

**Figure 4.**
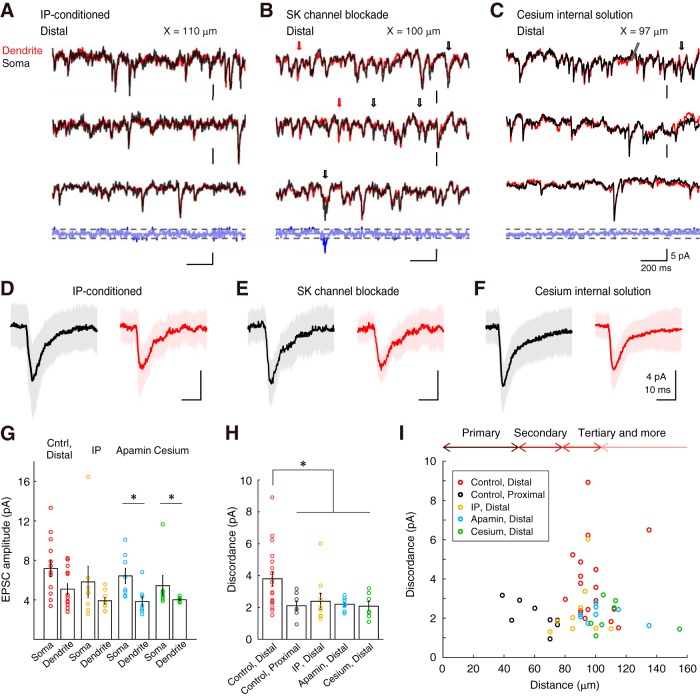
Disappearance of the dendritic heterogeneity via SK-channel downregulation. ***A***, Representative traces of sEPSCs in the paired recording from soma (black) and distal dendrite (red) after inducing IP. Subtraction of the third paired traces is shown at the bottom. The detection threshold was dotted. ***B***, Representative traces of sEPSCs under apamin administration. An arrow indicates a soma-dominant event. ***C***, Representative traces of sEPSCs under blockade of broad K^+^ channels by Cs^+^-containing internal saline. A double slash indicates a notch of traces. ***D***–***F***, Averaged sEPSCs from soma and distal-dendrite after IP conditioning, apamin administration, and cesium internal solution, respectively (black trace, soma; red trace, dendrite). Shading represents the SD (89–522 traces). ***G***, Bar graphs of EPSC amplitudes (IP: *n* = 8; apamin: *n* = 8, cesium: *n* = 7). *p < 0.05, two-tailed Mann–Whitney *U* test. Control data of the distal and proximal dendrites are the same as the data in Figure 3. ***H***, Discordance in sEPSCs compared between distal-, proximal-dendrites, IP-conditioned, and apamin-administered. *Significant difference of multiple comparison of HSD procedure following to one-way ANOVA at *p* < 0.002. ***I***, Discordance in the function of the distance. Colored circles show the discordance (pA) against distance on the branch from soma. Bidirectional arrows on the top indicate the corresponding ranked branch length obtained in Figure 2*F*. Discordance of EPSCs is prominent at farther than 80 μm, where the tertiary branching begins.

Our previous studies have suggested dendritic branches to have inhomogeneous excitability through the branch-specific modulation of ion channels (Ohtsuki et al., 2012b; Ohtsuki and Hansel, 2018). In the excitable dendrites after IP induction and SK channel blockade, the increase in the electrical conductance of the PF and CF inputs and back-propagated action potentials was manifested. In this study, to investigate whether sEPSC conductivity through dendrites is increased, I analyzed the sEPSCs after both the induction of IP (Fig. 4*A*) and the application of a SK channel blocker, apamin (10 nm; Fig. 4*B*). Even in the paired-recordings from the distal dendrites and soma, the time courses of voltage-clamp recording substantially corresponded to each other in the representative recordings of both IP-conditioned and apamin-administered cells (Fig. 4*A*,*B*). In fact, the subtraction of the soma and dendrite traces was nearly within the subthreshold level (±2 pA) in the representative recordings. These results suggest that the discordance of sEPSC in distal dendrites was suppressed by the SK channel blockade and an increase in electroconductivity by dendrites. To investigate the mechanism of the diminishment of the discordance in both IP-conditioned and apamin-administered conditions, I tested the level of discordance if broad K^+^ channels were blocked (Fig. 4*C*). As shown in the representative data using the Cs-based internal solution, instead of K-gluconate, the subtraction of the current traces of soma and dendrite was, again, nearly within the subthreshold level (Fig. 4*C*, bottom). This suggests that the electroconductivity of PC dendrites was enhanced by the blockade of broad K^+^ channels. I also showed averaged sEPSC waveforms on the soma and dendrites in both IP-conditioned and apamin-administered groups (Fig. 4*D–F*). Again, the sEPSC amplitudes in dendrites were smaller than that in the soma (Control Distal: rank sum = 214, *n* = 13, *p* = 0.0513; IP-conditioned: rank sum = 77, *n* = 8, *p* = 0.3822; Apamin: rank sum = 93, *n* = 8, *p = 0.0070; Cs-internal: rank sum = 69, *n* = 7, *p = 0.0379; two-tailed Mann–Whitney *U* test; Fig. 4*G*). Notably, although the difference between EPSC amplitude of soma and dendrite were ∼2 pA (Fig. 4*G*), the discordance of the sEPSC between the soma and dendrites in both IP-conditioned, SK-channel-blocked and Cs-internal solution groups were significantly lower than that in the control distal dendrites (*F*_(4,49)_ = 5.24, *p = 0.0015, one-way ANOVA with Tukey's HSD procedure; Fig. 4*H*). Therefore, despite the difference in sEPSC amplitude of soma and dendrite, the extent of discordance in distal dendrites is doubled. Additionally, the discordance in the distal dendrites was suppressed in IP-induced, apamin-administered, and cesium-loaded cells, suggesting that SK channels and IP may generate the sEPSC discordance in dendrites.

The relationship between the extent of sEPSC discordance and the distance of paired recording revealed that the increase in discordance is prominent from ∼80 μm in the distal dendrites (Fig. 4*I*). Morphological observation also indicated that the border where the discordance becomes prominent corresponded to the secondary bifurcation point of PC dendrites from the pooled data (Fig. 2). These results suggest that such discordance of the simultaneously recorded sEPSCs was dependent on the recording-location. This observation further implied that there is a limit to EPSC conduction in the PC dendrites at the distance of 80 μm. Moreover, the IP, SK-channel blockade, and internal cesium diminished the discordance between EPSCs, even at distal branches. Therefore, activity-dependent K^+^ channels, like SK channels, per se reduce the local excitability of the dendritic branches and limit the conduction of synaptic currents in dendrites, generating heterogeneous spaces of electrical conductivity.

### Clustered input on cerebellar Purkinje neurons

**Figure 5.**
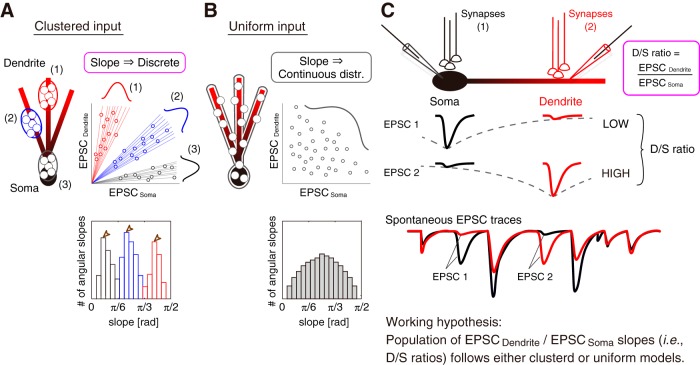
Deduction of a synaptic input pattern from the D/S ratio. ***A***, ***B***, Synaptic input hypothesis on PCs. I hypothesized two types of input patterns of active synapses from the distribution of the EPSC_Soma_ versus EPSC_Dendrite_ plot. ***A***, Clustered input: when synaptic activity is accumulated in several locations on the PC membrane, the distribution of the EPSC_Soma_ versus EPSC_Dendrite_ plot gathers to several clusters with distinct slopes, meaning that the slopes of co-EPSCs will form discrete distributions, as the assumed histogram below shows. ***B***, Uniform input: when synapses are activated at random locations, the slopes of co-EPSCs will form a continuous distribution as shown in the histogram below. ***C***, Slopes of co-EPSC events are defined as the ratios (i.e., EPSC_Dendrite_ divided by EPSC_Soma_) in this study. Regardless of either the amplitude of EPSC or the number of coincidently activated synapses, the D/S ratio is a function of the distance between the two recording positions. Paired-recorded spontaneous EPSCs include many EPSC events. Given the distribution of the population of D/S ratio follows either clustered or uniform models, the input pattern of co-EPSCs can be estimated.

Next, I asked how many groups of the discordance are observed in individual PCs. Given that PF terminals activity was suggested to be clustered during sensory processing (Wilms and Häusser, 2015), the population of ratios derived from individual co-EPSC events (i.e., EPSC_Dendrite_/EPSC_Soma_) are expected to accumulate into different modes when a bundle of functional synapses was activated repeatedly, regardless of the original EPSC amplitude in the local space (Fig. 5*A*). In contrast, if the activated synapses evoked sEPSCs in the random field on neuronal membranes, the relation between EPSC_Dendrite_ and EPSC_Soma_ would be uniformly distributed and its ratios would be contiguous (Fig. 5*B*). Henceforth, I refer to these co-EPSC ratios as D/S ratios. The advantage of such a rationale is in the absence of the consideration of EPSC amplitudes at each event (Fig. 5*C*).

**Figure 6.**
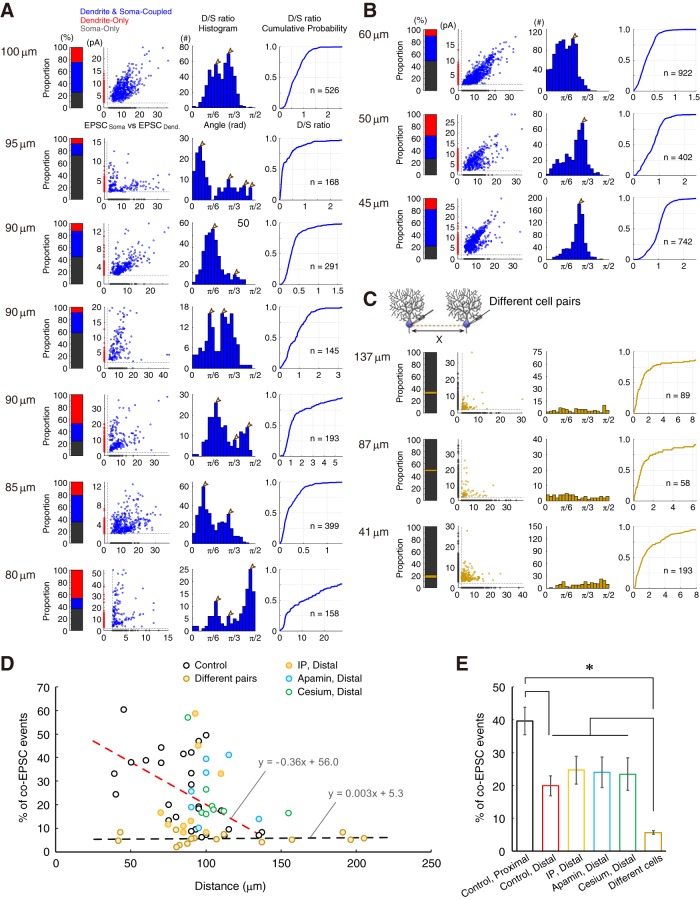
Inputs from a discrete population of granule cells to PC dendrites. ***A***, Seven representative analyses of sEPSCs paired-recordings from soma and distal dendrites (80–100 μm). Proportion of the total events, a plot of sEPSC amplitudes (*x*-axis: EPSC_Soma_, *y*-axis: EPSC_Dendrite_), a histogram of angular D/S ratio, and their cumulative probability with the number of co-EPSCs are shown. Blank arrowheads are peaks of clusters corresponding to Figure 7. Please note that the co-EPSC profiles display multiple nodes. ***B***, Co-EPSC profiles of three representative analyses of sEPSCs paired-recordings from soma and proximal dendrites (45–60 μm). ***C***, Co-EPSC profiles of three representative analyses of the sEPSCs from different neurons. Note that the *y*-scale in histograms of angular D/S ratios in ***A***-***C*** is adjusted to the entire EPSC data number. The maximum is set at ∼20% of data to make the difference visible. ***D***, Proportion of co-EPSC events in function of the distance: S-D or S-S. Dotted linear regression lines are added (red: Control, *r* = −0.559, *p* = 0.00163, *R*^2^ = 0.3122; black: Different pairs, *r* = 0.086, *p* = 0.760, *R*^2^ = 0.0074). ***E***, Bar graph of the proportion of co-EPSCs. *Significant difference of multiple comparison following to one-way ANOVA at *p* = 2.0e−6.

**Figure 7.**
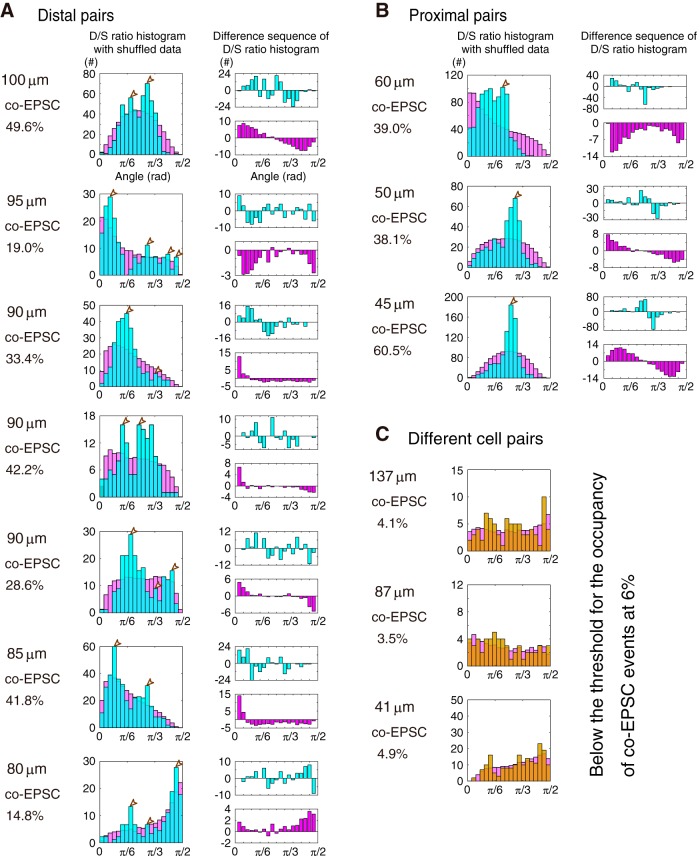
Representative analyses of the angular D/S ratios. The number of clusters is estimated from the D/S ratios of somatodendritic paired-recordings from PCs. All data in Figure 6*A–C* are shown. In each row, the distance between recordings, percentage of the co-EPSC events, histogram of the D/S ratios obtained from the EPSC_Soma_ versus EPSC_Dendrite_ plot (cyan) and the shuffled data (magenta) with a π/40 radian (4.5°) of the bin size, and histograms of difference sequence of the D/S ratio histogram (top, raw data; bottom, shuffled data) are presented. The peak of each cluster is marked by a blank arrowhead in the histograms of the angular D/S ratio. ***A***, Distal dendrite pairs. ***B***, Proximal dendrite pairs. ***C***, Different cell pairs. Please note the representative data of different-cell paired recordings with few co-events (at <6%-threshold). Details are described in Materials and Methods.

In Figure 6*A–C*, *I* displayed the representative data of the individual paired-recordings from the soma and both distal (Fig. 6*A*; 7 paired recordings, 80–100 μm) and proximal (Fig. 6*B*; 3 paired recordings, 45–60 μm) dendrites and from different somata (Fig. 6*C*; 3 paired recordings, 41–137 μm). Thereafter, sEPSC events were classified into the Dendrite and soma-coupled, Dendrite-only, and Soma-only by the analytical criteria and the proportion was shown in a stacked bar graph. Each sEPSC amplitude on soma and dendrite was plotted in scatter graphs. The resultant D/S ratios were displayed as both histograms and cumulative probability graphs, and related statistical analyses are shown in Figure 7.

With this analysis, I first found that the histograms of D/S ratios in distal dendrites showed multiple peaks (Fig. 6*A*, D/S ratio histogram), whereas those in proximal dendrites had substantially a single peak (Fig. 6*B*). This suggests that the D/S ratios form multiple clusters in distal dendritic recordings. Such clusters of the D/S ratio distribution are not obvious in the recording from different cell pairs (Fig. 6*C*), due to the small proportion of co-EPSC events (<6% on average). Next, I also found that the proportion of co-EPSC in total events was high in the recordings from the soma and proximal dendrite pair recordings, whereas it decreased in the distant paired-recordings (Fig. 6*D*); co-EPSCs detection in different-cell pairs was 5.7 ± 2.4% (*n* = 15, 41–205 μm) independent on the distance between pairs instead (*F*_(5,67)_ = 8.92, *p = 2.0e-6, one-way ANOVA with Tukey's HSD procedure; Fig. 6*E*). Results suggest that the proportion of co-EPSC is high in recording of soma and proximal dendrite, although it is low in recording of soma and distal dendrite. The decline of the co-EPSC proportion in the function of distance suggest the limit of EPSC electroconduction in dendrites. Therefore, EPSC in far distal dendrite may not correlate to the soma. Please note that the proportion of the co-EPSCs in somatodendritic recordings was significantly higher than the chance rate. Finally, its dependence on the distance indicates that the filtering effect of dendrites reduced the detection rate of simultaneous recordings of identical synaptic activity.

### A clustered synaptic-input model formed by intrinsic excitability of dendrites

**Figure 8.**
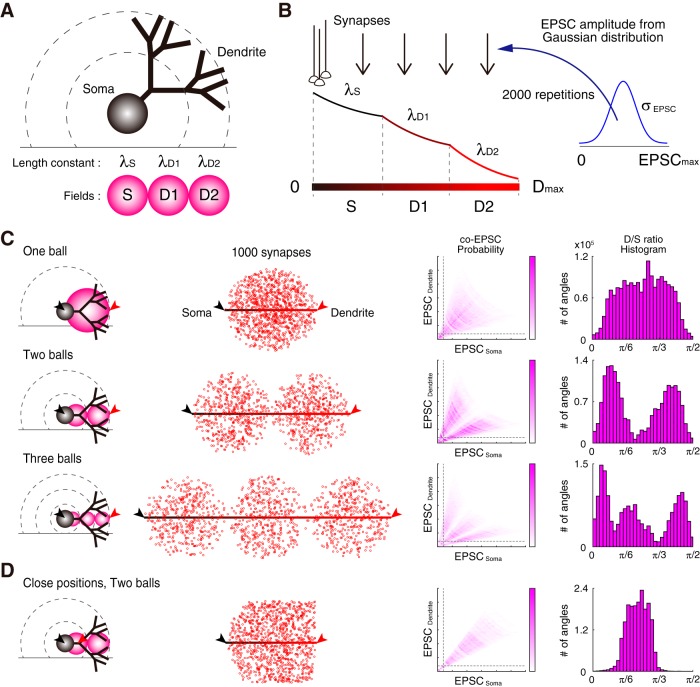
Concatenated-ball and multiple input-field models. ***A***, ***B***, To simplify the neuronal membrane fields, I compartmentalized them into multiple-fields, such as S, D1, and D2 in accordance with the three-ball-model (***A***). Each field is spherical and is arranged in tandem with the length constants: λ_S_, λ_D1_, and λ_D2_. At linear display of each concatenated field with a different length constant is shown in ***B*** (0 to D_max_) (Rall, 1962). Considering synapses formed around the soma at point 0, the EPSC amplitude is obtained randomly from the Gaussian distribution within *N*(2σ, σ^2^) in each trial, and the EPSC amplitude decreases along the attenuation function with each length constant at the field. Please note the locations of 1000 synapses were selected randomly in the entire fields of the model. Successively, I obtained the EPSC amplitude of every synapse on the soma (at 0) and dendrite (at D_max_), respectively. I repeated the trial for 2000 times and obtained both the averaged probability in the co-EPSC coordinate and the simulated angular D/S ratio histogram as the summation of repetitions. Details are described in the Materials and Methods. ***C***, Concatenated-ball models. Synapses given an EPSC amplitude are randomly distributed in the fields: one ball, two balls, and three balls. Each sample image of synaptic locations is displayed in the next figures to the right. Soma–dendrite axes are expanded from concatenated-ball models for visibility. EPSC amplitudes calculated on soma and dendrite are plotted as EPSC_Soma_ versus EPSC_Dendrite_. The simulated angular D/S ratios are represented as histograms at a bin size of π/40 radian with distinct modes. ***D***, Same simulation with a two-ball, short distance of paired somatodendritic recording. With short S-D distance, the histogram of D/S ratio has a single peak.

**Figure 9.**
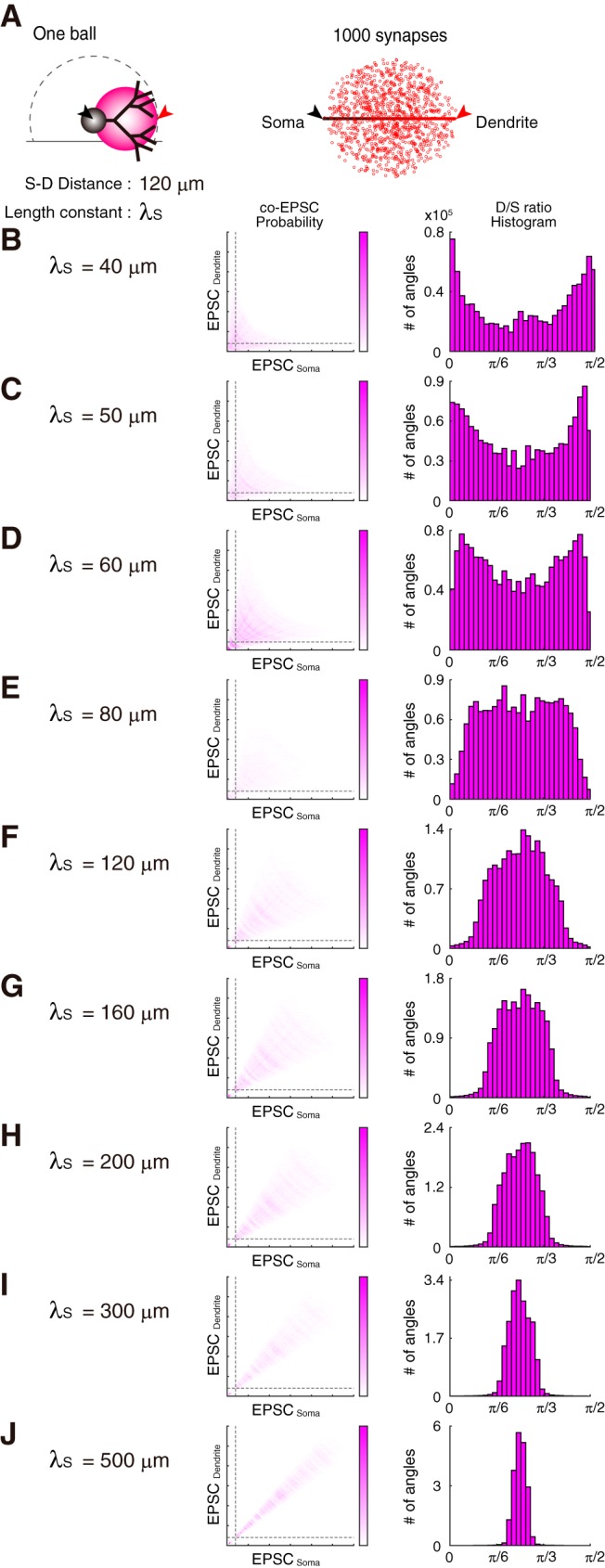
Sample analyses of the single-ball model. The neuronal membrane field of one-ball model was applied a uniform length constant, λ_S_: 40–500 μm. One thousand synapses with randomly selected EPSC amplitude from the Gaussian distribution were distributed on the membrane spherical area, and EPSC amplitudes on both of the soma and dendrite were obtained (***A***). I obtained co-EPSCs 2000 times, and averaged co-EPSC probability and the accumulated histograms of the number of D/S ratio angles were displayed in ***B***–***J*** with various length constants. The distribution of the D/S ratio angles narrows in increasing the length constant.

Regarding the number of peaks in the D/S ratio histograms, it is generally not easy to count the number of modes from a histogram drawn by obtained data, without knowing the original distribution. Before the estimation of the number of co-EPSC clustering, I tested whether spatial patterning of synaptic activity may warrant the distribution of co-EPSC. To do so, I made a simple simulation of the soma-dendrite process with several concatenated spheres to express the neuronal membrane fields (Figs. 8, 9). The membrane fields were separated as the soma and different dendrites, while co-EPSC probability and D/S ratio histogram was obtained in one- to three-ball models (Fig. 8*C*,*D*). As depicted in the co-EPSC probability plots, densely colored regions of probability were emerged based on the number of balls, indicating a correlation between the number of clustered fields of synaptic inputs and the distribution of D/S ratios. Furthermore, I created a model reflecting the paired-recordings from two close positions (Fig. 8*D*). Even with the two-ball model, the histogram showed a single peaked distribution of the D/S ratio angles, similarly to the recording samples between soma and proximal dendrites in Figure 6*B*. In addition, the distribution of the D/S ratio angles becomes narrower in increasing the length constant (Fig. 9). Compared with the results of the simply concatenated-ball model, the clustered field of sEPSC can explain the distinct pattern of distribution of D/S ratios. Both my simulation and co-EPSC analyses supported the idea that synaptic inputs on the soma and dendrites may form distinct components in the basal state.

**Figure 10.**
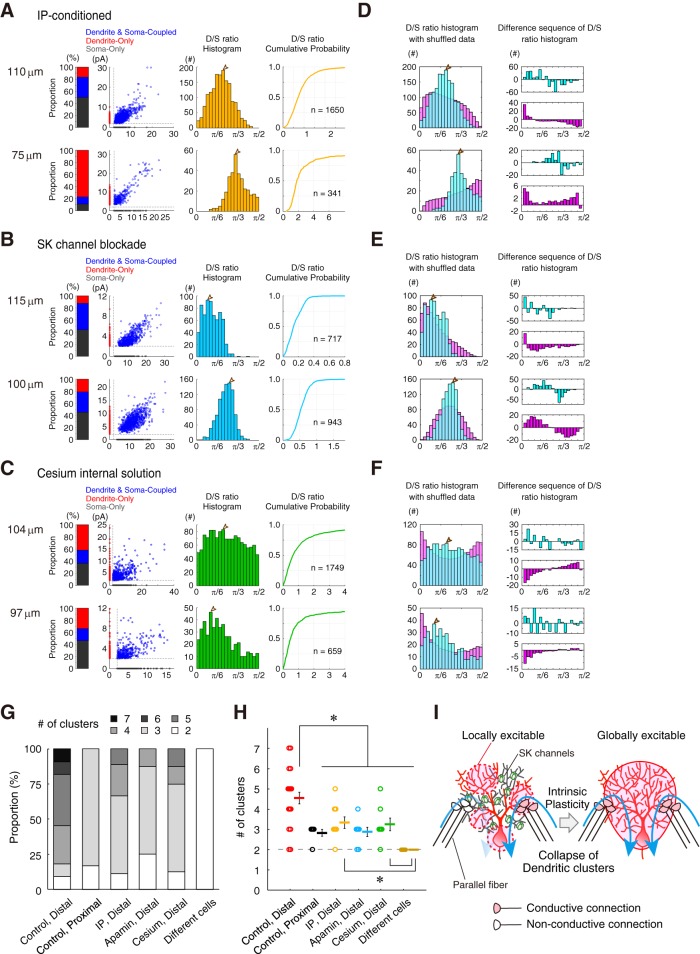
Modification of synaptic clustering via the intrinsic excitability plasticity. ***A***–***C***, Representative analyses of sEPSC from soma and distal dendrites after IP conditioning (***A***), apamin administration (***B***), and cesium permeation (***C***). The proportion of the total events, a plot of sEPSC amplitudes, a histogram of the angular D/S ratio, and the cumulative probability with the number of co-EPSCs are reported. Blank arrowheads, peaks of clusters as in Figure 7. ***D***, IP-conditioned distal dendrite pairs. Histograms of D/S ratio (cyan) with shuffled data (magenta) are shown. ***E***, SK-channel-blocked distal dendrite pairs. ***F***, Cs-loaded cells' distal dendrite pairs. ***G***, Proportion of cells and the associated number (#) of clusters in six experiments. Please note that the estimated number of clusters include Dendrite-only and Soma-only groups. ***H***, Mean ± SEM of the number of clusters. *Significant difference of multiple comparison of the HSD procedure following to one-way ANOVA at *p* = 1.8e−9. ***I***, A cellular learning model related to the collapse of dendritic clusters. Collapse of dendritic clusters by IP related to a cellular learning model.

Last, I identified the number of clusters from the data of 68 paired-recordings after a selection based on the co-EPSCs proportion using a threshold of the statistical power (1-β > 0.8; see Materials and Methods). Of these, 11 data from different-cell pairs were < 6% of co-EPSCs. And, 11 pairs were found to be without clustering from the criteria of the statistical power. Histograms of angular D/S ratios (Fig. 7) described 90.9% (20/22) of the recordings from distal dendrites have more than one mode (ranged from 1 to 5: i.e., 3–7 clusters; Fig. 10*G*), suggesting that neurons presented at least one cluster in angular D/S ratios of co-EPSC events. Additionally, such PCs were shown to present 4.5 ± 0.3 clustered fields of excitatory inputs, when the Dendrite- and Soma-only groups were included in the analyses (Fig. 10*G*,*H*, Control, Distal). In contrast, none (0/6) of the paired recordings from proximal dendrite had more than one mode (i.e., 2 or 3 clusters; Fig. 10*G*,*H*, Proximal), indicating that the sEPSC events observed in distal dendrites arise from differently excitable fields among branches. The results from different-cell pairs without clustering support the different analyses in recordings from identical cells (Fig. 10*G*,*H*).

Next, I investigated whether the IP and SK-channel blockade modulated the D/S ratio distribution. I analyzed the co-EPSC events recorded after IP induction, under apamin and cesium administration (Fig. 10*A–C*), respectively. Both IP-conditioned, apamin-administered and cesium-loaded neurons showed 6 of 25 (24.0%) multiple-peaked distributions of the D/S ratios [IP-conditioned: 33.3% (3 of 9 cells), SK-channel blockade: 12.5% (1 of 8 cells), broad K-channel blockade: 25.0% (2 of 8 cells)], suggesting that IP and SK-channel blockade are related to the spatial clustering of sEPSC seen on PC dendrites (Fig. 10*G*), although the amount of clustering was significantly different among the dataset (*F*_(5,67)_ = 14.65, *p = 1.8e-9, one-way ANOVA with Tukey's HSD procedure; Fig. 10*H*). Moreover, my results imply that SK channels may generate functionally compartmentalized fields on neurons. Therefore, sEPSC of substantial PCs in the cerebellum shows clustering along the soma-dendrite axis *in vitro* in a temporal detection rate. Further, my data indicated the clustering of the neuronal surface fields to emerge due to the activity-dependent suppression of the conduction by SK channels. The different excitability of dendrites segregates synaptic inputs conductive or less-conductive, while IP changed the clustered fields into a global activation via the SK channels downregulation (Fig. 10*I*). However, there was a minor gap of the discordance (Fig. 4*H*) and the estimated number of clusters (Fig. 10*G*,*H*) in the comparison of SK-channel blockade by apamin with IP-conditioned or cesium internal. The IP conditioning of 5 Hz-depolarization might be less effective to change the dendritic excitability than apamin administration due to less invasion of the depolarization to far distal dendrites (i.e., space clamp failure). Cesium invasion is also concerned not to achieve to the fine processes of far distal dendrites. The drive of transporters may weaken the effect of blockade of K^+^ channels, too.

Typically, investigators have used a cesium-based pipet solution to enhance space clamp from cerebellar PCs. The findings shown here may suggest that using cesium would eliminate the physiological differences of distal dendrite with soma. Less functional clustering was observed in cesium internal (Figs. 4*C*,*F*, 10*C*,*H*). This would be an important message of this study and of value to demonstrate further evidence.

## Discussion

In this study, I recorded sEPSC on soma and dendrite, simultaneously. First, I found that EPSCs recorded on distal dendrite were uncorrelated to those recorded on soma (Fig. 3*A*,*G*), suggesting a limited electroconduction. This was not observed in the proximal dendrites, but such discordance emerged at farther than secondary bifurcations. The discordance of EPSCs in distal dendrite was abolished by IP-conditioning and SK-channel blockade, indicating the dendritic filtering via SK-channel functions (Fig. 4). Ratio analyses (Figs. 6, 10) and simple branch-field models (Fig. 8) confirmed that PCs have more than four distinct groups of PF input. Furthermore, the estimated clustering was collapsed by IP and SK-channel blockade.

### Branch-specific modulation of the intrinsic excitability underlies the clustered synaptic inputs

The synaptic potentials on the PF terminal to distal PC dendrites had been considered to achieve merely short distances, given the attenuation through dendrites processes (De Schutter and Bower, 1994; Roth and Häusser, 2001). It is true that Na^+^ action potentials generated around the initial segment of the soma do not actively propagate into dendritic branches (Stuart and Häusser, 1994; Ohtsuki et al., 2012b; Yamamoto et al., 2019), partially because of a low Na^+^ channel density in PC dendrites (de Ruiter et al., 2006). Therefore, PC dendrites were considered to lack a boosting mechanism necessary for the back-propagation (Stuart and Häusser, 1994; Palmer et al., 2010) and, seemingly, the synaptic activity, too. However, considering the localization of characteristic voltage-gated Na^+^ channels, which generate the resurgent currents (Na_V_ 1.2 and Na_V_ 1.6 subunit on PC dendrites; de Ruiter et al., 2006), such resurgent Na^+^ channels may also contribute to the dendritic excitability (Raman and Bean, 1997; Lewis and Raman, 2014; Shim et al., 2018). Recent studies on the heterogeneous excitability among dendritic branches, through mechanisms underlain by BK, SK, and A-type K^+^-channels (Rancz and Häusser, 2006; Ohtsuki et al., 2012b; Otsu et al., 2014; Ohtsuki and Hansel, 2018; Zang et al., 2018), argue against the uniform pattern of the intrinsic excitability of dendritic branches. In addition, the modulation of the intrinsic excitability through *Ih* channels was also found in this neuron (Nolan et al., 2003; Loewenstein et al., 2005; Yang and Santamaria, 2016). Among ion channels, SK channel function on PC excitability has been relatively well examined. The blockade of SK channels on distal dendrites drastically changed the firing pattern (Womack and Khodakhah, 2003). This may have resulted from the disruption of the heterogeneous dendritic excitability, which regulates active firing patterning. Findings in this study support the scenario of heterogeneity of the dendritic excitability. The results suggest that the IP and SK-channel blockade abolished such the branch-specific difference of electroconductivity (Fig. 4*I*). Further, PC dendrites and its plasticity of the intrinsic excitability suggest modifying the gating ability (i.e., electroconductivity). By reducing the conduction of minor weak inputs on distal dendrites, PC dendritic branches may segregate them to either pass or fail.

This study also suggests the existence of heterogeneity among postsynaptic branch fields and a resultant clustering of the postsynaptic currents, caused by the restriction of the electric conduction through dendrites (Figs. 6, 10). One study demonstrated clustered presynaptic activity, as hot spots, at PF synapses in response to sensory stimulation (Wilms and Häusser, 2015). Considering that the number of synapses which PF varicosities make onto PC dendrites is ∼1.05 in rats (Harvey and Napper, 1991), the original granule cells that evoked the clustered activity on PF terminals would be different cells among distinct population of the granule cells. Importantly, the firing frequency of granule cells in response to sensory stimulation is suitable for the induction of branch-specific excitability plasticity, similar to our bursting protocol for IP induction of 5 Hz PF stimulation (Ohtsuki et al., 2012b). Thus, the population activity of granule cells projected from a circuit with certain functions can change the sensitivity of PC dendritic arbors.

### Expected mechanisms of the regulation of synaptic current conduction

In present study, I postulate two explanations behind the modulation mechanism of the D/S ratio distribution and sEPSC clusters. First, SK channels may suppress the excitability of the local branch fields. In response to both elevation of Ca^2+^ concentration and voltage change (Adelman et al., 2012), SK channels suppress the local fields excitability on dendritic branches and spines (Faber et al., 2005; Ngo-Anh et al., 2005). In cerebellar PCs, T-type Ca^2+^ channels with a low voltage threshold for their activation (∼−65 mV) mediate the Ca^2+^ entry into spines in response to the subthreshold synaptic input, activating SK2 channels (Womack et al., 2004; Ly et al., 2016). Under blockade of T-type Ca^2+^ channels, a series of PFs stimulation elongate EPSP waveforms (similar to those under a SK channel blocker). Therefore, PFs can suppress the conduction of synaptic current to the neighboring field via a shunting effect. In contrast, SK-channel downregulation plausibly improves the conduction through branch fields.

The second explanation is the elongation of the cable conduction. Generally, length constant is defined as the rate of a negative single exponential function. It depends on the square root of the ratio between membrane resistance and internal resistance of the neural cable. Herein, I expected that the downregulation and blockade of SK channels increased the membrane resistance and that the length constant would be elongated. However, as shown in our previous study and present data, the input resistance of PCs soma and dendrites did not change substantially after IP-induction and SK-channel blockade (Ohtsuki et al., 2012b), suggesting slender changes in the length constant. Therefore, results suggest that the branch-specific shunt of SK-channel gating was unleashed by IP, rather than the change in cable conduction. This SK-channel modulation may broaden the area of EPSCs transmission by a release from the activity-dependent suppression (Fig. 10*I*).

### Functional significance of the intrinsic plasticity and its branch specificity

Branch-specific modulation of the membrane excitability was found to accompany with mechanisms including the downregulation of SK channels in PCs (Ohtsuki et al., 2012b; Ohtsuki and Hansel, 2018). Compared with the plasticity of synaptic efficacy of PF-LTP, the absolute impact of the SK-channel modulation was large enough to evoke additional action potentials when activating a few bundles below threshold. Therefore, the modulation of intrinsic excitability per se has an ability to modulate the synaptic weight per dendritic arbor with multiple modalities among the branch fields, manifesting the conduction paths (Fig. 10*I*). That may represent another learning or memory storage mechanism at a cellular level (Zhang and Linden, 2003; Cai et al., 2004; Frick et al., 2004; Magee and Johnston, 2005; Campanac et al., 2008; Ito, 2008; Larkum et al., 2009; Ohtsuki et al., 2009, 2012b; Adelman et al., 2012; Ohtsuki and Hansel, 2018; Wang et al., 2018). Although I focused on the SK-channel downregulation via IP in this study, different activity-dependent K^+^ channels can regulate the dendritic clustering. Possibly, the cellular computational power would be stronger in neurons with well compartmentalized dendrites as recently suggested in the human brain (Beaulieu-Laroche et al., 2018).

Immune activation is another trigger to modulate the excitability of PCs in both their firing frequency and dendrite excitability (Yamamoto et al., 2019). Activation of the microglia increased the firing frequency and the intrinsic excitability of the PC dendrites. Effect of inflammation-induced hyperexcitability of PCs modulated the rodents' behaviors. Therefore, abusing the modulation of intrinsic excitability and dendritic compartments may explain the animals' behaviors at the foundation of the cellular physiology.

There are two types of PC-specific KO mice, deficient for molecules involved in the IP induction: PP2B and STIM1 (stromal interaction molecule 1), both of which showed IP failure. They have been analyzed the motor coordination and learning of eye movements and the eye-blink conditioning (Schonewille et al., 2010; Ryu et al., 2017). Then, the PP2B-KO mice showed defects in the vestibulo-ocular reflex (VOR) and its adaptation. PP2B-KO mice also showed impairment of the eye-blink conditioning (Schonewille et al., 2010). The other transgenic mice, STIM1-KO, showed a deficit in the memory consolidation of the VOR adaptation (Ryu et al., 2017). Therefore, the IP and branch excitability are suggested involved in the motor performance. Interestingly, an *ex vivo* PCs from animals experienced delay eye blink conditioning showed the reduction of after-hyperpolarization and occlusion of the IP-induction, even after 48 h later of the learning paradigm (Titley et al., 2018), suggesting the conditioned memory had been maintained not only in the cerebellar nuclei (Wang et al., 2018), but also in cerebellar cortex as the modulated excitability engram. Another study suggests lower excitability in the zebrin/aldolase C-positive PCs, through TRPC3 channels, which produces different subgroups among the cerebellar PCs (Zhou et al., 2014). Thus, it would be interesting if such difference contributes the functional clustering in PC dendrites.

In sum, the heterogeneous expression pattern of SK channels on dendrites (Belmeguenai et al., 2010) may give rise to the functional clustering on PC branch fields. Because the PC dendrites show complex branching and active electro-responsiveness, the specific location of afferent synapses may influence the response pattern, memory storage, and animal behaviors via branch-specific IP induction (Masoli and D'Angelo, 2017).
